# Potent Vasodilator and Cellular Antioxidant Activity of Endemic Patagonian Calafate Berries (*Berberis microphylla*) with Nutraceutical Potential

**DOI:** 10.3390/molecules24152700

**Published:** 2019-07-25

**Authors:** Camila Calfío, Juan Pablo Huidobro-Toro

**Affiliations:** 1 Laboratorio de Farmacología, Departamento de Biología, Facultad de Química y Biología, Universidad de Santiago de Chile, Santiago 9170022, Chile; 2 Centro para el Desarrollo de Nanociencia y Nanotecnología (CEDENNA), Universidad de Santiago de Chile, Santiago 9170022, Chile

**Keywords:** Calafate (*Berberis microphylla*) extract, vasodilator potency, antioxidant activity, glycosylated anthocyanins, glycosylated flavonols

## Abstract

Hydroalcoholic extracts of Patagonian Calafate berry (*Berberis microphylla*) contain mono or disaccharide conjugated anthocyanins and flavonols. The Liquid Chromatography-Mass Spectrometry (LC-MS) chemical extract profile identified glycosylated anthocyanidins such as delphinidin-, petunidin- and malvidin-3-glucoside as the major constituents. The predominant flavonols were 3-O substituents quercetin-rutinoside or -rhamnoside. Anthocyanins doubled flavonols in mass (13.1 vs. 6 mg/g extract). Polyphenols vascular actions were examined in the rat arterial mesenteric bed bioassay; extract perfusion elicited concentration-dependent vasodilatation mimicked by conjugated anthocyanins standards. Vascular responses of main glycosylated anthocyanins were endothelium-dependent (*p* < 0.001) and mediated by NO production (*p* < 0.05). The anthocyanins antioxidant activity determined in isolated endothelial cells (CAA) showed a reduced redox potential as compared to the extract or quercetin. While in the 2,2-Diphenyl-1-picrylhydrazyl (DPPH) assay, the anthocyanins showed an equivalent quercetin potency, the extract was 15-fold less active, proposing that the anthocyanin-induced vasodilation is not due to an antioxidant mechanism. The extract shows promising commercial nutraceutical potential.

## 1. Introduction

The consumption of berries and fresh fruits promotes health due to the high content of antioxidant polyphenols associated with cardiovascular disease protection [1]. The main polyphenols reported in berries are flavonoids (anthocyanins, flavonols, flavanols), tannins and phenolic acids [2]; these chemicals are potent antioxidants with additional antimicrobial, anti-inflammatory and vascular properties [3,4,5]. Among the red-blue variety of berries, Calafate (*Berberis microphylla*) an endemic Patagonian plant, has an outstanding profile since it is reported to have the highest antioxidant profile among the endemic and commercial Chilean fruits [6]. Chemically, Calafate berries contain high levels of phenolic compounds such as anthocyanins, flavonols and phenolic acids plus ascorbic acid [7,8,9,10,11]. Despite the ample chemical characterization of Calafate fruits including its redox potential, its pharmacological properties have been scarcely investigated. Ethnomedical reports indicate that Calafate leaves and roots have antibiotic properties [12]; however, little is known about the vasodilator properties of the fruit’s components excepting for its anti-inflammatory effect [13,14] or its use nutritional value by Patagonian people.

Recently Calfio, Mateluna, and Huidobro-Toro [15] demonstrated that a hydroalcoholic Calafate berry extract has vasodilator activity, but its mechanism remains elusive. Based on the Calafate fruit chemical profile and on its high polyphenol content, including glycosylated anthocyanins, we proposed to investigate whether the antioxidant activity of the berries constituents is somehow related to its vasodilator response. In addition, we were interested in assessing whether nitric oxide (NO) production participates in the vasodilatation in view that NO is a potent endogenous vasodilator synthesized by endothelial nitric oxide synthase (eNOS). This enzyme, typically expressed in endothelial cells, is an essential cardiovascular regulator that participates in the sec-to-sec vascular wall homeostatic control [16]. The endothelium is currently regarded as a dynamic tissue which lines the entire vascular system modulating vascular function through the synthesis of several physiologically relevant vasoactive factors including dilators such as NO or peptide vasoconstrictors. Moreover, since glycosylated anthocyanins are the main fruit polyphenols, we became particularly interested in examining the proposal that anthocyanins, as present in the fruit, are more potent vasodilators than other flavonoids studied [17,18,19] and that the vasodilator effect of anthocyanins is NO-mediated. This hypothesis could be somehow be related to an increased NO bioavailability elicited by polyphenols associated with their antioxidant activity. In this regard, flavonoids could prevent eNOS uncoupling avoiding tetrahydrobiopterin (BH_4_) oxidation [16], or reduce NO-inactivation by superoxide, peroxynitrite or another cellular ROS [20,21]. Alternatively, these compounds could elicit a direct activation of eNOS activity in endothelial cells to increase NO production.

To this aim, the arterial rat mesenteric bed was chosen as a vascular bioassay since this vascular territory contains both conductance and resistant vessels, a suitable model to study vascular actions and at the same time quantify NO production released to the mesentery perfusate following extract or standard flavonoids applications. In addition, we determined antioxidant properties in endothelial cells harvested from the rat mesenteries to examine the relevance of Patagonian Calafate berry as a functional food. Moreover, this is the first study that characterizes the vasodilator effects of Calafate extract and established that its vasodilator activity is not necessarily linked to its antioxidant properties.

## 2. Results

### 2.1. Chemical Characterization, Identification and Relative Quantification of the Main Anthocyanins and Flavonols Extract Constituents

**Figure 1:**
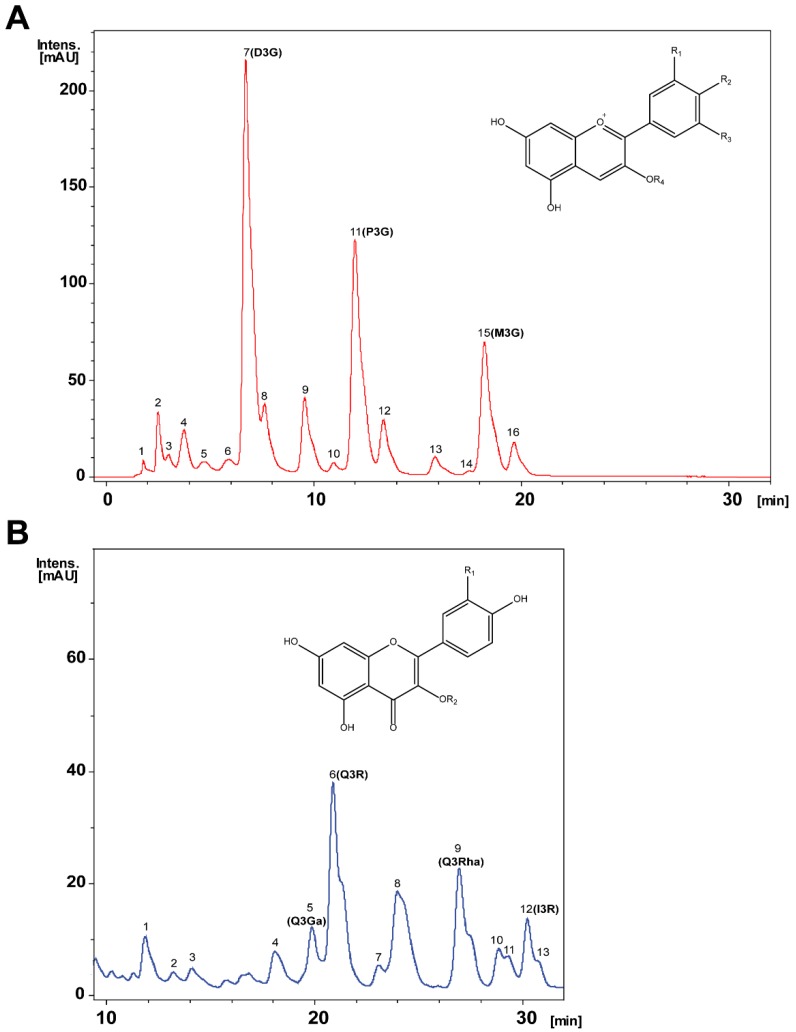
Representative chromatographic analysis of Calafate berry extracts. (**A**) Prototype anthocyanins chromatogram of the hydroalcoholic extract based on 520 nm absorbance. Sixteen major peaks were observed; mass spectra analysis of the peaks revealed the list of putative compounds shown in Table 1. Inset shows the basic anthocyanin structure; position 3 conjugations are referred to as R_4_ substituents. (**B**) Representative chromatogram of hydroalcoholic extract flavonols identified by absorbance at 360 nm; the 13 major peaks identified and analyzed by mass spectrum are listed in Table 1. The basic flavonols structure and its glycosylated substituents in R_2_ refer to the various sugar conjugates present in the extract.

Chromatographic profiles of the Calafate hydroalcoholic berry extract identified 16 anthocyanins (Figure 1A, and structural chemical frame) plus 13 flavonols (Figure 1B including basic chemical structure). Chemical identity was assigned based on UV-vis and MS spectra of the peaks listed in Table 1. Anthocyanin peaks 7–11, 13 and 15 matched identities with pure commercial standards to confirm assigned identity. The three major peaks correspond to delfinidin-3-glucoside (7, D3G), petunidin-3-glucoside (11, P3G) and malvidin-3-glucoside (15, M3G). Likewise, flavonols were similarly examined; out of the 13 peaks identified, peak 5 corresponded to quercetin-3-galactoside (Q3Ga), peak 6 to quercetin-3-rutinoside (Q3R), peak 9 to quercetin-3-rhamnoside (Q3Rha), peak 12 to isorhamnetin-3-rutinoside (I3R); confirmed using commercial standards. Table 1 lists the chemical characterization data.

Based on chemical identity (Figure 1A,B plus Table 1), relative peak quantification of the Calafate hydroalcoholic extract constituents was achieved using external standards from commercially pure flavonoids. Delphinidin-3-glucoside was the main extract anthocyanin followed by petunidin-3-glucoside and malvidin-3-glucoside. Table 2 lists each Calafate chemical constituent amount expressed as mg/g extract; minor anthocyanins present in the extract were also determined (Table 2). The main extract flavonol was quercetin-3-rutinoside followed by quercetin-3-rhamnoside and isorhamnetin-3-rutinoside. Altogether, the Calafate extract contained twice as much anthocyanidins compared to flavonols (13 versus 6 mg/g extract, Table 2). Other constituents were not quantified.

### 2.2. Comparative Studies between the Vascular Response Elicited by the Calafate Extract was Mimicked by the Main Calafate Extract Flavonoids; Effects in the Rat Arterial Mesenteric Bed Bioassay

**Figure 2:**
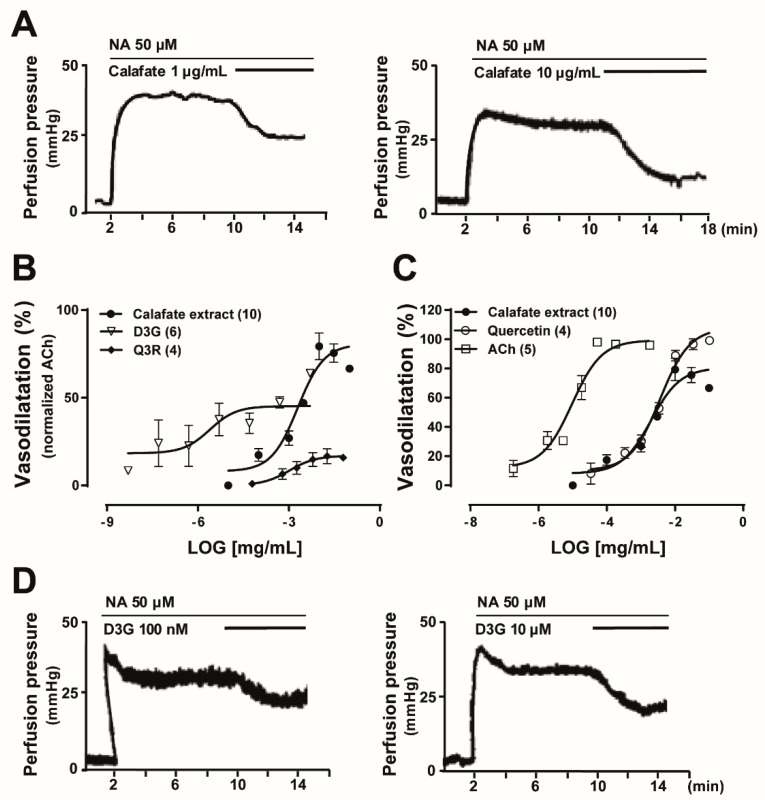
Concentration-dependent vasodilatation induced by Calafate berry extracts and the mimetic action of delphinidin-3-glucoside, quercetin, quercetin-3-rutinoside or acetylcholine in the rat arterial mesenteric bed. (**A**) Representative tracings of the hydroalcoholic extract-induced vasodilatation elicited by perfusion with 1 or 10 µg/mL; the extract was dissolved in distilled water and diluted in perfusion buffer. (**B**) The normalized 1 µM ACh concentration-dependent vasodilatation curves of the hydroalcoholic extract (solid circles) were compared to the main anthocyanin (delphinidin-3-glucoside, D3G open triangle) or flavonol (quercetin-3-rutinoside, Q3R solid diamond). (**C**) Comparison of the vasodilatation elicited by the Calafate extract, quercetin (open circles) or acetylcholine (ACh, open square). Symbols represent the mean values; bars the SEM. The number in parenthesis of panels B and C represents protocol replicates. (**D**) Representative tracings of the vasodilator response induced by 100 nM and 10 µM delphinidin-3-glucoside (D3G). All the experiments were made in intact mesenteric beds (with endothelium).

Intact pre-contracted rat mesenteries were perfused with dilutions of the hydroalcoholic Calafate berry extract; concentration-dependent vasodilatations were recorded (Figure 2); the vasodilator EC50 was 3.72 ± 0.9 µg/mL. Its Emax reached 80.6 ± 5.1% (Figure 2B,C), normalized with 1 µM ACh used as an internal control. In 7/10 experiments Emax occurred with 10 µg/mL; values ranged from 75%–98%. The extract prepared with acetone, purposely planned to extract slightly more hydrophobic compounds, also caused concentration-dependent vasodilatations, with an EC50 of 8.82 ± 2.5 µg/mL; Emax ranged from 57%–90%. These findings suggest that both extract constituents must be similar, chemical characterization using LC-MS analysis of the acetone extract further supported this assumption (data not shown).

To compare whether the vasodilator extract response was mimicked by its constituents, we examined separately the vasodilator activity elicited by commercial D3G, P3G or M3G, Q3R or Q3Rha. D3G elicited a potent and concentration-dependent vasodilator response, which was at least 100-fold more potent than the extract (Figure 2B). Notwithstanding, D3G perfusion reached only a 54.8% ± 4.5% of the ACh normalization (Figure 2B). Moreover, perfusion with 100 nM P3G or M3G elicited vasodilator responses of the same magnitude as 100 nM D3G (Table 3 and Figure 2D). Q3Rha, the second most predominant Calafate berry flavonol (Table 2), required 10 µM to elicit a vasorelaxation comparable to 100 nM D3G or the other glycosylated anthocyanins (Table 3), indicating that this flavonol was 100-fold less potent than the anthocyanins. Q3R, elicited concentration-dependent vasodilator responses that reached a maximal response of 18.8% ± 2.4% (Figure 2B), suggesting that the disaccharide was less potent and less efficacious compared to its parent monosaccharide.

In addition, ACh, quercetin and the hydroalcoholic Calafate extract showed parallel concentration-response curves (Figure 2C) all reaching over 80% Emax. The quercetin concentration–response curves almost superimposed with the Calafate extract, both curves differ in the Emax value (Figure 2C). ACh and quercetin Emax values are identical (99.4% ± 4.1% vs. 99.9% ± 3.2%).

### 2.3. Calafate Extract and Glycosylated Anthocyanins Antioxidant Properties

**Figure 3:**
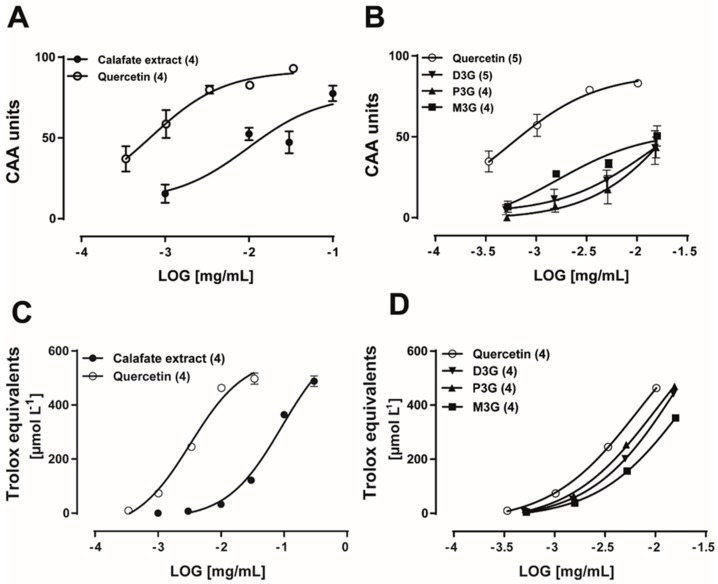
Comparison of the antioxidant activity of the extract and major extract anthocyanins by two methods: the cellular antioxidant activity (CAA) versus the DPPH assay. (**A**) Calafate extract and quercetin control antioxidant concentration–response curves. (**B**) Antioxidant effect of the three major Calafate anthocyanins in the CAA assay measured in endothelial cells examined in the same concentration range that caused vasodilatation. Calafate extract antioxidant activity measured by the DPPH assay and its comparison with quercetin (**C**) or the three major extract anthocyanins (**D**). Symbols represent the mean values, bars the SEM. While the CAA activity is expressed in relative units, the DPPH assay is expressed in Trolox equivalents.

The antioxidant activity of the extract and its standards was evaluated by two assays. The extract CAA EC50 was 32.4 ± 0.02 µg/mL; its Emax was 77.5 ± 4.8 CAA units, while the quercetin EC50 was 0.54 ± 0.0003 µg/mL (n = 4, Figure 3A), its Emax was 93 ± 1.7 CAA units, indicating that quercetin was 60-fold more potent as an antioxidant than the extract in the endothelial cell assay. The anthocyanins D3G, P3G and M3G showed, at the largest concentration examined (15 µg/mL), an Emax similar for the three compounds, which averaged 43.3 ± 9.4, 43.4 ± 6.4, 50.6 ± 6.2 CAA units, respectively (Figure 3B), indicating a low antioxidant potential in this assay. The extract antioxidant activity examined in the DPPH assay showed a similar profile being markedly less potent than quercetin (Figure 3C). In the DPPH assay, glycosylated anthocyanins showed an antioxidant potential like that of quercetin (Figure 3D). Altogether, the present findings establish that the antioxidant profiles examined in CAA cells vs. DPPH assay are significantly different. Moreover, it is possible to infer that the vasodilator activity of glycosylated anthocyanins is not likely related to their antioxidant potential in endothelial cells.

### 2.4. The Calafate Extract and Main Glycosylated Anthocyanins Elicit Endothelium and NO-Mediated Vasodilatations

In view that 10 µg/mL of the hydroalcoholic Calafate extract consistently caused about 85% vasodilatation, this concentration was chosen to characterize its mechanism of action and will be referred as the extract in the next protocols.

**Figure 4:**
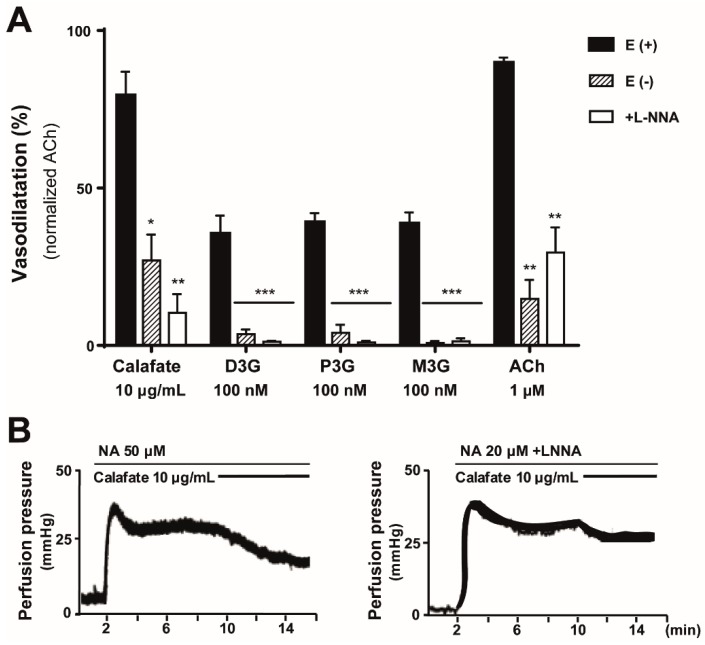
Endothelium-dependent mechanism of the Calafate extract and anthocyanin vasodilation; endothelial nitric oxide synthase enzyme (eNOS) participation. (**A**) The vasodilatation elicited by 10 µg/mL extract (n = 10) or 100 nM of delphinidin-3-glucoside (D3G, n = 6), petunidin-3-glucoside (P3G, n = 6), malvidin-3-glucoside (M3G, n = 4), or acetylcholine (ACh, n = 19) perfused in intact mesenteries or by the same mesentery devoid of endothelium (-). In parallel, separate protocols evaluated the role of eNOS activity after enzyme blockade following tissue perfusion with 150 µM L-NNA. Vasorelaxations were normalized by the 1 µM ACh-induced vasodilation. Columns denote the mean values, bars the SEM. *, *p* < 0.05, **, *p* < 0.01, ***, *p* < 0.001 (**B**) Representative tracings of the vasodilatation elicited by 10 µg/mL Calafate extract before (control) and following eNOS inhibition with L-NNA. Note that after L-NNA treatment, the concentration of NA was reduced to 20 µM, because of tissue sensitization.

The Calafate extract vasodilation in mesenteries devoid of the endothelial layer was reduced by 73% ± 8.2% (Figure 4, *p* < 0.05), revealing a preponderant endothelium-dependence of this response. Likewise, the vasodilatation elicited by perfusion with 100 nM D3G, P3G or M3G was abolished completely (Figure 4, *p* < 0.001). To assess whether NO participates in the vasodilatation, eNOS activity was inhibited using L-NNA; this procedure significantly reduced the vasodilatation induced by the extract (89.6% ± 5.8%, *p* < 0.01) or the three anthocyanins (98.7%–99.3%, *p* < 0.001). These results are summarized in Figure 4 and suggest that NO mediates the vascular response. In all cases, 1µM ACh was used as an internal control; its vasodilator activity was significantly reduced by endothelium denudation or eNOS inhibition by L-NNA (Figure 4).

**Figure 5:**
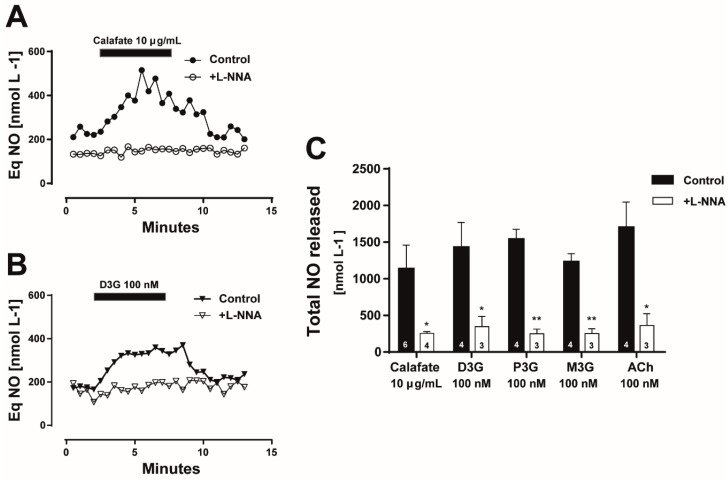
Luminally accessible NO determinations elicited by Calafate extract perfusion or the main anthocyanins. (**A**) Representative time course of NO released to the perfusate following 10 µg/mL Calafate extract application (closed symbols) and following eNOS blockade with 150 µM L-NNA (open symbols) in the same preparation. (**B**) Representative time course of luminally accessible NO elicited by perfusion with 100 nM of delphinidin-3-glucoside (D3G) in a control preparation (closed symbols) and following L-NNA (open symbols). (**C**) Statistical analysis of NO production elicited by perfusion with 10 µg/mL Calafate extract, 100 nM delphinidin-3-glucoside (D3G), petunidin-3-glucoside (P3G), malvidin-3-glucoside (M3G) and acetylcholine (ACh) before and after treatment with the enzyme inhibitor. Columns represent the mean values; bars the SEM. The number inside the columns represents protocol repetitions in separate mesenteries. *, *p* < 0.05; **, *p* < 0.01.

Moreover, in the next step, we quantified the NO production in intact vascular beds with and without eNOS inhibition. Perfusion with the Calafate extract elicited an increase in the luminally accessible NO which was not observed in the same tissues pre-treated with the eNOS inhibitor; a representative time course of the luminally accessible NO released is illustrated in Figure 5A. Perfusion with D3G, the main extract anthocyanin, showed a similar time course of NO production, which was annulled following eNOS inhibition (Figure 5B); the quantification of these results are summarized in Figure 5C. The NO released by the extract and the corresponding anthocyanins standards were significantly blocked by L-NNA treatment (*p* < 0.05); in all cases, the NO production was proportional to the vasodilator response (data not shown). As an internal control, tissue perfusion with 1 µM ACh also elicited a rise in the luminally accessible NO, which was significantly attenuated by L-NNA pre-treatment (Figure 5C, *p* < 0.05). Overall, these findings strongly support that NO is the main intracellular messenger of the Calafate extract vasodilator responses or the anthocyanins induced vasodilatations suggesting that the extract constituents are putative eNOS targets. While both the Calafate extract and anthocyanins increase luminal NO production, they apparently differ in the kinetics of NO production. The rise in the luminally accessible NO elicited by the extract was slower than that caused by D3G; moreover, the anthocyanin-induced rise in NO reached a maximal response that is better maintained than that of the extract. This finding could be related to the multiple bioactive extract compounds that likely mask the individual anthocyanin response.

## 3. Discussion

The total flavonols and anthocyanins content of the hydroalcoholic Calafate berry extract was comparable to previous reports, minor differences were found, likely attributed to fruit growth, harvest or ripening [8,11], evidencing a chemical fingerprint independent of the Calafate growth latitude. Moreover, the acetone extract yielded similar results, confirming the main constituents. Consistent with a similar chemical profile, the vasodilator activity of the hydroalcoholic or acetone Calafate extracts were similar and related to concentration- and endothelium-dependent vasodilator responses of the rat arterial mesenteric bed via a NO-dependent mechanism.

The study of the Calafate berry antioxidant properties seemed straight forward in view of its reported antioxidant profile among endemic and commercial Chilean fruits, reaching 25,662 µmol Trolox equivalents/100 g fresh weight [6]. As expected, the extract antioxidant activity reported, based on the DPPH-scavenging assay plus the CAA bioassay, recognize and support this potential. Previous studies with other chemicals detailed that the in vitro DPPH-scavenging activity is not equivalent to the CAA examined in different cell types [22,23,24]. Endothelial cells were chosen for CAA determinations to match the extract vasodilator activity and to gain further insights into the in vivo extract activity. A novelty of this strategy reveals that the antioxidant potential of glycosylated anthocyanins in the bioassay is less than in the in vitro chemically-based assay, suggesting glycosylated anthocyanins do not contribute largely to the in vivo extract antioxidant potential. This difference may be due to the pharmacokinetic variables required for the CAA bioassay, such as membrane solubility, endogenous cell antioxidants, diffusion barriers, among other variables [22]. Moreover, the present results validate that in the rat mesentery, glycosylated anthocyanins antioxidant profile does not account for the vasodilatory potencies. Likely, other extract polyphenols or constituents may contribute to the total berry redox potential.

A most remarkable observation refers to the difference in the pharmacological potency and efficacy of the Calafate extract as compared to the anthocyanins or flavonols standards. The present results reveal the nanomolar vasodilator potency of delphinidin, petunidin or malvidin-3-glucosides compared to structurally related flavonoids [25,26]. In addition, the vasorelaxation elicited by conjugated extract anthocyanins is exclusively endothelium-dependent [15], while quercetin vasodilates by a dual mechanism. The quercetin response is probably associated with an endothelial mechanism [26] plus a preponderant smooth muscle action [25,27,28], likely related to potassium channel opening and smooth muscle cell hyperpolarization [25,27]. In addition, blockade of voltage-gated calcium channels, or decrease in intracellular calcium levels [27], are mechanisms that may also be involved at the smooth muscle level. We infer that the extract anthocyanins may target eNOS activity to promote NO production by an undetermined mechanism. This proposal is supported by a significant attenuation of the extract vasodilatation by either endothelium removal or eNOS blockade; the remaining component may be attributed to vascular smooth muscle relaxation.

Regarding the nanomolar potency of glycosylated extract anthocyanins to directly influence NO production, we infer that either anthocyanins diffuse into the cell to activate somehow eNOS, or signal through a cell membrane pathway. The log D partition coefficient of D3G at pH 7.0 is 1.35 as compared to −0.78 for its aglycone, a finding that discards a simple diffusion process through the endothelial cell membrane. Therefore, these compounds may either be metabolized by the tissue to the corresponding aglycones or the glycosylated species permeate the endothelial cell membrane by a carrier [29,30], such as a glucose transporter [31,32]. Alternatively, based on structural similarities with the phytoestrogen genistein, the high anthocyanin vasodilator potency may reflect a high affinity membrane binding site, such as the α estrogen receptor (ERα) [33], thought to elicit a signaling pathway to increase NO production, mimicking the 17-β-estradiol effects [33] likely via a non-genomic mechanism. In support of this contention, delphinidin increased several pathways that favor NO production such as Src, ERK1/2 and eNOS Ser1177 phosphorylation. Moreover, in transgenic mice aorta lacking the ERα, or using siRNA or ERα antagonists, the vasodilator delphinidin effect was abolished [33]. In addition, cyanidin-3 glucoside increased Akt activity and eNOS phosphorylation, both enzymes directly involved in NO production [34], suggesting that both aglycones and the conjugated anthocyanins elicit NO production intimately associated to endothelium-dependent vasorelaxation.

By comparing the vasodilator activity of the Calafate extract with anthocyanins or flavonols, two further assumptions arise (i) D3G is 1000-fold more potent than the hydroalcoholic extract but half less efficacious, (ii) Q3R is markedly less potent and less efficacious than D3G and obviously less potent compared to the extract activity. We deduce from these observations that the extract must contain other active biomolecules that mask the D3G potency and increase its efficacy, as well as other constituents to increase the potency of conjugated flavonols. Conjugated anthocyanins and flavonols concur to account for the extract activity in intact mesenteries, with endothelium; synergism might occur since multiple constituents may be at play. In addition, we repeatedly observed that upon perfusion with 100–300 µg/mL, the motor responses induced by NA were reduced reversibly by 29.7% ± 1.7% (n = 4), supporting that the extract contains, in addition, an α-adrenergic blocking compound or a smooth muscle relaxant.

In summary, the Calafate extracts have both vasodilator activity and antioxidant potential; the combination of these two properties support the elaboration of a nutraceutical with beneficial value for chronic vascular disease treatments, as a solid biotechnological projection of these novel findings.

## 4. Materials and Methods

### 4.1. Animals

Adult male Sprague Dawley rats 45 days old (200–250 g) bred at the Animal Reproduction Facility of the Faculty of Biological Sciences of the P. Catholic University of Chile were used throughout. NIH (USA) standards for animal breeding and handling care were strictly adhered to. The Universidad de Santiago Ethical Committee for the use of animals in biological research approved the specific protocols designed for this research (776) and supervised our strict adherence to the subscribed procedures. The Faculty of Chemistry and Biology University plus the Ethical Committee of the supervised project development; every possible effort to reduce the number of sacrificed animals was enforced.

### 4.2. Calafate Berry Extract Preparation

Calafate berries were collected from the Temuco area (38°S, Chile) in Jan 2015; fruits were kept frozen at −80 °C until performing extractions. Pools of berries were lyophilized; 80 g of dried fruits were pulverized in a mortar and macerated in ethanol/water/acetic acid (50:49.5:0.5 *v*/*v*), referred as hydroalcoholic extract or prepared as an acetone extract (acetone/water/acetic acid, 50:49.5:0.5 *v*/*v*). These homogenates were constantly stirred for 24 h at room temperature in darkness. After, the soluble portion was separated by centrifugation; the solid residue was extracted two consecutive times until color was removed with each solvent mixture. The soluble portion was concentrated to dryness under reduced pressure at 40 °C followed by lyophilization for its usage in biological and chemical protocols. The resultant extracts were stored at −20 °C; chemical and biological stability were maintained for up to two years after extract preparation, as assessed by a comparable antioxidant and/or vasodilator activity recorded during research progress. This procedure achieved 23.5% ± 0.7% extraction efficiency.

### 4.3. Chemical Identification of the Extract by LC-MS

Ten milligram of the extracts was dissolved in 1 mL of milliQ water and filtered through a Polyvinylidene difluoride (PDVF) membrane (0.22 μm). To separate the extract constituents, 20 μL of the extract was injected to an LC-MS system (HPLC Agilent 1100 (Agilent Technologies Inc., Santa Clara, CA, USA) coupled to Electrospray ionization ion trap (ESI-TRAP) Esquire 4000 (Bruker Daltonics GmbH, Germany). HPLC was monitored by ChemStation for LC 3D Rev. A.10.02 (Agilent Technologies Inc., Santa Clara, CA, USA) program and the esquireControl 5.2 (Bruker Daltonics GmbH, Bremen, Germany) the mass spectrometer.

The identification of the extract anthocyanins and flavonols was performed following the method for Calafate flavonoids [8], slightly modified since a C18(2) Luna column 150 × 4.6 mm, 5 µm and 100 Å (Phenomenex Inc., Torrance, CA, USA) was used. The column exit split the flow towards the UV detector and the mass spectrometer. Chromatography used a mobile phase gradient of water/acetonitrile/formic acid (87%:3%:10% *v*/*v*/*v*) (solvent A) and water/acetonitrile/formic acid (40%:50%:10% *v*/*v*/*v*) (solvent B); at a flow rate of 0.8 mL min^−1^. The phase program was 94% to 85% solvent A for 10 min, 85% to 70% for 15 min, 70% to 50% for 5 min, 50% to 94% in 1 min and 9 min stabilization to 94%. The ionization process was made at 3000 V assisted for nitrogen as the nebulizer and desolvation gas at 50 psi, 10 L/min flow and 365 °C. The chromatogram and mass spectra were acquired with electrospray ionization in positive and negative modes; the extract anthocyanins were identified by 520 nm while flavonols at 360 nm wavelength. The compounds identity assignment was based on HPLC-MS chromatography, comparing the extract components with a laboratory database mass spectrum plus UV-vis, which included previous published chemical characterization data of Calafate berry extracts [7,8,9,11]. Moreover, the identity of eight of the anthocyanins plus four flavonols was confirmed using commercial standard comparisons. The analytical characterization of the hydroalcoholic extract constituents is listed in Table 1.

### 4.4. Quantification of the Principal Glycosylated Anthocyanins and Flavonols from the Calafate Extract

Extract anthocyanin and flavonol quantification was performed in a Waters Alliance 2695 system equipped with a W2690/5 separation module unit, a DAD detector, and a 4.6 × 150 mm, 5 μm C18 Waters Spherisorb column at 40 °C. The chromatographic separation was carried out using the same gradient system that LC-MS. Quantification was achieved by external standardization, using pure commercial standards of glycosylated anthocyanins and glycosylated flavonols purchased from Extrasynthese (Genay, France). Stock solutions of all standard anthocyanin compounds were prepared in methanol-HCl 0.1% while flavonols in ethanol. Flavonoids calibration protocols were prepared by diluting stock solutions in ranges of 0.01 to 0.2 mM; calibration curves plotted peak areas versus concentrations (R^2^ = 0.99). Results are expressed as milligrams of compound per gram extract, as shown in supplemental Table 2.

### 4.5. Vascular Reactivity Assays and Quantification of Vasodilator Responses

Adults male Sprague-Dawley rats were anesthetized by intraperitoneal ketamine (75 mg/kg)-xylazine (5 mg/kg). The abdominal cavity was excised in the midline; the superior mesenteric artery was cannulated to start mesenteric bed perfusion [35,36,37] via a peristaltic pump (2mL min^−1^) with Krebs-Ringer buffer solution (equilibrated with 95% O_2_, 5% CO_2_) at 37 °C. Mesenteries were excised from the intestinal wall and placed in a dish designed to collect the perfusion fluid [38]. A pressure transducer was connected at the entrance of the superior mesenteric artery and connected to a Grass polygraph recorder to register the changes in perfusion pressure elicited by the extract. The mesenteric preparation was stabilized for 20 min by perfusion buffer solution before starting experiments; pressure fluctuations were interpreted as changes in the resistance of the arterial mesenteric network. To analyze vasodilatation, the arterial bed was pre-contracted with 50 µM noradrenaline (NA) to increase the perfusion pressure by 30–45 mmHg, a condition that favors vasodilation assays. Every single protocol started by using 1 µM acetylcholine (ACh) used as a control to assess endothelium indemnity. For the bioassays, the extract lyophilizate was reconstituted in distilled water (50 mg/mL) and diluted thereafter in perfusion buffer.

Vasodilatations were quantified as the percentage reduction of the NA-induced contractions by application of either 0.1–300 μg/mL of extracts or 1nM–10 μM of standard flavonoids used to perform concentration–response curves. All protocols used at least four separate rats to determinate concentration–response curves. In cases, the same rat was used to assess more than one protocol. Vasodilator responses are expressed as the percentage of dilatation of each extract or compounds concentration tested, which were individually normalized by the 1 µM acetylcholine response elicited by each mesentery. Concentration–response curves were adjusted to sigmoid curves using Graph Pad Prism; potency (EC50) and the maximal pharmacological efficacy (Emax) were derived from each bioassay.

### 4.6. Denudation of the Endothelial Cell Layer of the Mesenteric Bed

To evaluate the role of the endothelium in the vascular response elicited by 10 µg/mL extract, mesenteries were perfused with 0.1% saponin for 90 s to remove a substantial portion of the endothelial layer without damaging the adjacent vascular smooth muscle; this procedure was previously reported Boric et al. [38]. Endothelial damage was evidenced by a substantial reduction of the ACh-induced vasorelaxation as compared to its response recorded prior to endothelium removal. Results are expressed as a percentage of the extract or ACh induced dilatation before and after endothelium denudation. At least 4 separate mesenteric preparations to examine this protocol.

### 4.7. eNOS Blockade by Structural L-Arginine Analogues Reduce the Extract-Induced Vasodilation

The participation of eNOS was evaluated in mesenteries before and after 1 h tissue incubation with 150 µM Nω-nitro-L-arginine (L-NNA) before and after tissue perfusion with 10 µg/mL of the extract. Following endothelium removal, the NA concentration used to contract the tissues was generally reduced from 50 to 3 µM, due to the excessive vasomotor response elicited in the absence of endothelium. The extract vasodilator response was evaluated as before. NO release was determined in non-contracted mesenteries, to avoid interference due to NO release triggered by NA vasoconstriction [38]. To determine the luminally accessible NO released to the media, perfusate samples were collected every 30 secs, 2 min before, during the 6 min extract perfusion and for additional 5 min after extract application, to examine whether NO basal levels recovered upon discontinuing extract perfusion. A similar protocol was designed to examine extract-induced NO release in mesenteries pre-treated with 150 µM L-NNA. In all cases, at least 4 separate rat mesenteries evaluated this protocol.

### 4.8. Determination of the Extract and Anthocyanins Cellular Antioxidant Activity (CAA) in Endothelial Cell Derived from the Mesenteric Bed

CAA determinations used endothelial cells from the rat mesentery. Primary cultures from the mesentery were isolated as reported Donoso et al. [39]; the cell pellet obtained was resuspended in supplemented growth medium 199 (ECGS 2% *v*/*v*, fetal bovine serum 20% *v*/*v*). These cells were maintained for 5 h at 37 °C and 5% CO_2_, then the medium was removed, and the cells were washed with phosphate buffer saline. Thereafter, cells were incubated with fresh media until reaching 80% confluence; cells were next trypsinized and seeded at a density of 2.5 × 10^4^/well on a 96-well black plate with a clear bottom in 100 µL of growth medium and incubated for 48 h at 37 °C. For CAA determinations, the protocol designed by Wolfe and Liu (2008) was adapted for endothelial cells with slight modifications. After 48 h of cell growth, cells were incubated for one hour with 100 µL of 199 media supplemented with 150 µM of L-NAME (L-NG-Nitroarginine methyl ester), a competitive eNOS inhibitor to avoid interferences due to cellular NO production. Immediate thereafter, cells were washed twice with phosphate buffer and co-incubated with 1–100 μg/mL of the hydroalcoholic Calafate extract or 1–30 μM anthocyanin or quercetin used as an internal control plus 15 μM of fluorescein diacetate (dissolved in cell media) for additional 20 min. Although the figure shows extract or flavonols amounts in mg/mL, the equivalent molarities were also calculated. The cells were rinsed again with phosphate buffer to remove the extracellular fluorescein and added immediately with 125 μM ABAP (2,2′-azobis (2-methylpropionamidine) dihydrochloride) in 100 µL Hanks balanced salt solution (HBSS). Each assay consisted of triplicate internal controls, blanks and extract diluted samples, standard anthocyanins or quercetin used as a control for these concentration-dependent determinations. All experiments were conducted using endothelial cells isolated from at least 4 rats; extract lyophilizate and flavonoids were dissolved in distilled milliQ water and diluted in cell media; a 60 mM quercetin stock was prepared DMSO and later diluted in cell media.

Fluorescence was quantified in a Tecan infinite 200 pro equipment (Tecan Trading AG, Switzerland) at 37 °C every 5 min for 2 h (excitation at 485 nm and emission at 538 nm). The fluorescence versus time area under the curve was integrated to determinate CAA values for each treatment as described by Wolfe and Liu [24]. Results were compared with the quercetin standard curve used as a control. In view that the preponderant target of extract constituents is linked to a vasodilator response, which occurs in endothelial cells, we did not consider the antioxidant potential of the extract in smooth muscle cells.

### 4.9. Antioxidant Activity Quantified by the DPPH-Scavenging Assay

The extract free radical scavenging capacity or the standard anthocyanins and quercetin were determined by the DPPH assay as previously described [40], with minor modifications. This assay is considered the in vitro golden standard of antioxidant activity. The assay was performed in 96 well plates; 200 µL of a 150 µM DPPH solution prepared daily in 80% ethanol/water (*v*/*v*)) was added to all wells except for the blanks. Extract or standard flavonoids concentration-response curves were prepared by adding 25 µL of a stock sample solution added to the corresponding wells. The extract final dilution reached 1–300 µg/mL while 1–100 µM or equivalent mg/mL for the flavonoids. The plate was gently mixed for 10 s, covered and maintained in a dark place at room temperature for 2 h. Thereafter, the absorbance for the well was detected at 515 nm using the TECAN reader. These assays used 100–500 µM Trolox as an internal control to generate a calibration curve (R^2^ > 0.98); results are expressed as Trolox equivalents.

### 4.10. NO Determinations and Quantification

NO was quantified by chemiluminescence using a Seviers 280 NOA analyzer (GE Analytical Instruments, Boulder, CO, USA) as previously detailed [37]. In brief, perfusate samples were injected into the reaction chamber of the NOA equipment which contained 100 mg of potassium iodide in 8 mL of glacial acetic acid bubbled with nitrogen as a carrier of the NO produced. This reaction reduced the nitrites in the perfusate sample to NO, which emitted chemiluminescence generated by the NO–ozone reaction. The response was proportional to the nitrite equivalents in the media. Calibration of the equipment was routinely performed using sodium nitrite as a standard in concentrations ranging within three orders of magnitude, from 10 nM to 10 μM. The results were expressed either as the time course of NO equivalents (nmol/L) or as the total NO released above basal values (nmol/L). The area under the curve was calculated of the NO peak released by the extract.

### 4.11. Statistical Analysis

Data analysis and graph designs were performed using GraphPad Prism version 6.0 software program (San Diego, CA, USA). Analysis of variance (ANOVA) with post Bonferroni test and Student’s t-test were used to compare and determinate the statistical difference between the groups. Statistical significance probability was set at α < 0.05.

### 4.12. Chemical Compounds used in this Research

Commercial anthocyanin and flavonol standards were purchased from Extrasynthese (Genay, France). Acetylcholine (PubChem CID: 6060), noradrenaline (PubChem CID: 439260), L-NNA (PubChem CID: 440005), L-NAME (PubChem CID: 39836), saponin (PubChem CID: 137322043), ABAP (PubChem CID: 76344), DPPH (PubChem CID: 2735032) and Trolox (PubChem CID: 40634) were purchased from Sigma-Aldrich (St Louis, MO, USA). Fluorescein diacetate (PubChem CID: 65047) was purchased from Thermo Fischer Scientific (Waltham, Mass, USA). Analytical grade buffer salts and solvents were obtained from Merck (Darmstadt, Germany).

## Figures and Tables

**Table 1:** Anthocyanins and flavonols identified in the hydroalcoholic Calafate extract following LC-MS analysis.

Peak ^a^	Retention Time (min)	Molecular Ion	Product Ions			Analyte
**Anthocyanins Identified**						
1	2.0	641.2	479	317		Petunidin-O-hexoside-O-hexoside
2	2.8	627.1	464.9	303		Delphinidin-O-hexoside-O-hexoside
3	3.1	611.2	449	303		Delphinidin-rhamnosylhexoside
4	3.7	641.6	479	317.2		Petunidin-O-hexoside-O-hexoside
4	3.9	595.4	449	286.9		Cyanidin-O-rhamnosylhexoside
5	4.7	787.2	625.1	479.1	317.2	Petunidin-O-rhamnosylhexoside-O-hexoside
5	5.0	448.7	286			Cyanidin-O-hexoside
6	5.5	655.2	493	331.2		Malvidin-O-hexoside-O-hexoside
6	6.0	462.4	300.1	269		Peonidin-O-hexoside
6	6.4	655.4	493	331.1		Malvidin-O-hexoside-O-hexoside
7 ^b^	6.9	465.0	303			Delphinidin-O-hexoside
8 ^b^	7.8	611.5	465	303		Delphinidin-O-rhamnosylhexoside
9 ^b^	9.6	449.2	286.9			Cyanidin-O-hexoside
10 ^b^	11.0	595.6	448.9	286.9		Cyanidin-O-rhamnosylhexoside
11 ^b^	12.3	479.0	317			Petunidin-O-hexoside
12	13.7	625.1	478.9	317		Petunidin-O-coumaroyl-O-hexoside
						Petunidin-O-rhamnosylhexoside
13 ^b^	15.8	463.7	301			Peonidin-O-hexoside
14	17.4	464.0	300.9			Peonidin-O-hexoside
15 ^b^	18.9	493.1	331.1			Malvidin-O-hexoside
16	19.7	639.5	493	331.1		Malvidin-O-rhamnosylhexoside
Flavonols Identified						
1	12.0	477.4	314.8			Isorhamnetin-O-hexoside
2	13.1	623.3	314.9			Isorhamnetin-O-rutinoside
3	14.9	479.5	316.8			Myricetin-O-hexoside
4	19.0	464.0	300.7			Quercetin-O-hexoside
5 ^b^	19.8	463.1	300.7			Quercetin-O-hexoside
6 ^b^	20.7	609.4	300.7			Quercetin-O-rutinoside
7	22.9	505.6	462.9	300.8		Quercetin-O-acetyl hexoside
8	24.2	505.5	462.9	300.7		Quercetin-O-acetyl hexoside
9 ^b^	27.2	447.1	300.7			Quercetin-O-rhamnoside
10	28.7	537.5	374.9			Biapigenin
11	29.6	537.2	374.8			Biapigenin
12 ^b^	30.2	623.4	314.9			Isorhamnetin-O-rutinoside
13	30.8	478.5	314.8			Isorhamnetin-O-hexoside

^a^ Peak number according to the identified compounds in the Calafate hydroalcoholic extract. ^b^ Calafate extract constituents identity confirmed using commercial standards.

**Table 2:** Major flavonoids quantified by high-performance liquid chromatography with diode array detection (HPLC-DAD) in Calafate hydroalcoholic extract.

Peak	Chemical Standards	Chemical Substituents	mg/g Extract
**Main Anthocyanins**			
7	**D3G**, Delphinidin-3-glucoside	R_1_: OH, R_2_: OH, R_3_: OH, R_4_: Glucose	4.04 ± 0.02
8	**D3R**, Delphinidin-3-rutinoside	R_1_: OH, R_2_: OH, R_3_: OH, R_4_: Rutinose	1.23 ± 0.02
9	**C3G**, Cyanidin-3-glucoside	R_1_: OH, R_2_: OH, R_4_: Glucose	0.92 ± 0.01
10	**C3R**, Cyanidin-3-rutinoside	R_1_: OH, R_2_: OH, R_4_: Rutinose	0.44 ± 0.02
11	**P3G**, Petunidin-3-glucoside	R_1_: OH, R_2_: OH: R_3_: OCH_3_, R_4_: Glucose	3.81 ± 0.03
13	**Po3G**, Peonidin-3-glucoside	R_1_: OCH_3_, R_2_: OH, R_4_: Glucose	0.44 ± 0.02
15	**M3G**, Malvidin-3-glucoside	R_1_: OCH_3_, R_2_: OH, R_3_: OCH_3_, R_4_: Glucose	2.24 ± 0.04
**Main Flavonols**			
1	**Q3Ga**, Quercetin-3-galactoside	R_1_: OH, R_2_: Galactose	0.84 ± 0.01
2	**Q3R**, Quercetin-3-rutinoside	R_1_: OH, R_2_: Rutinose	2.05 ± 0.02
3	**Q3Rha**, Quercetin-3-rhamnoside	R_1_: OH, R_2_: Rhamnose	1.53 ± 0.01
4	**I3R**, Isorhamnetin-3-rutinoside	R_1_: OCH_3_, R_2_: Rutinose	1.49 ± 0.02

**Table 3:** Vasodilatation (%) elicited by 100 nM anthocyanins or 10 µM flavonols in the isolated rat arterial mesenteric bed with intact endothelium.

Concentration	Compounds	n	Vasodilatation (%) Mean ± SEM
100 nM	Delphinidin-3-glucoside, **D3G**	4	43.50 ± 8.0
100 nM	Petunidin-3-glucoside, **P3G**	6	39.08 ± 2.9
100 nM	Malvidin-3-glucoside, **M3G**	4	38.65 ± 3.5
10 µM	Quercetin-3-rhamnoside, **Q3Rha**	4	30.83 ± 1.99
10 µM	Quercetin-3-rutinoside, **Q3R**	4	14.83 ± 3.92
100 nM	Acetylcholine, **Ach**	5	66.95 ± 8.25
